# Limited choice of natural amino acids as mimetics restricts design of protein lysine methylation studies

**DOI:** 10.1038/s41467-023-39777-8

**Published:** 2023-07-11

**Authors:** Sara Weirich, Albert Jeltsch

**Affiliations:** Institute of Biochemistry and Technical Biochemistry, University of Stuttgart, Allmandring 31, 70569 Stuttgart, Germany

**Keywords:** Transferases, Cell signalling, Methylases, Methylation

## Abstract

Protein lysine methylation plays important biological roles but its experimental characterization is limited by the lack of suitable mimetics of methylated and unmethylated lysine among the natural amino acids. Here, we summarize the consequent challenges and discuss alternative approaches for biochemical and cellular lysine methylation studies.

Protein methylation mainly occurs on lysine and arginine residues^1, 2^. It is observed on histone and non-histone proteins and plays important roles in normal cellular physiology and diseases like cancer^3–5^. Protein methylation often regulates protein/protein interactions^4, 6^; in many cases so-called reader proteins are involved which further mediate downstream biological effects^7^. Depending on the reader protein, methylation can be required for binding to the target protein or it can inhibit binding. However, protein methylation is incompletely understood: for most protein methyltransferases (PMTs) the full spectrum of substrate proteins is not defined, for many identified protein methylation sites the responsible PMT enzyme has not yet been discovered, and for many protein methylation events their readers and biological effects are unknown.

Studies characterizing the roles of protein post-translational modifications often make use of amino acid substitutions to try to mimic the modified or unmodified protein state. In the kinase field, S-to-E exchanges are used as phosphomimic mutations. This approach is plausible as the oxygen atoms in glutamate are placed equally to the phosphoryl-group oxygens in phosphoserine, although glutamate lacks a third oxygen and it does not fully mimic the polarity of the phosphate ester (Fig. 1a). Hence, proteins with phosphorylated serine altered to glutamate often are bound specifically by the corresponding phosphoserine readers and S-to-E mutations often trigger similar conformational changes of the host protein as serine phosphorylation, although this needs to be validated in each case. There are some other cases, in which natural amino acids can be used as a proxy for modified ones although the similarity of the physicochemical properties is less pronounced. Examples include using glutamate as mimic of phosphothreonine or glutamine as mimic of acetyllysine (Fig. 1a, b, see for example^8^). In these applications, conclusions need to be validated very carefully. However, direct amino acid mimetics of methylated amino acids residues (or their stably unmethylated counterparts) are missing among the natural amino acids which limits experimental approaches in the protein methylation field. Similar difficulties preclude a straightforward investigation of many other protein modifications.

**Fig. 1:**
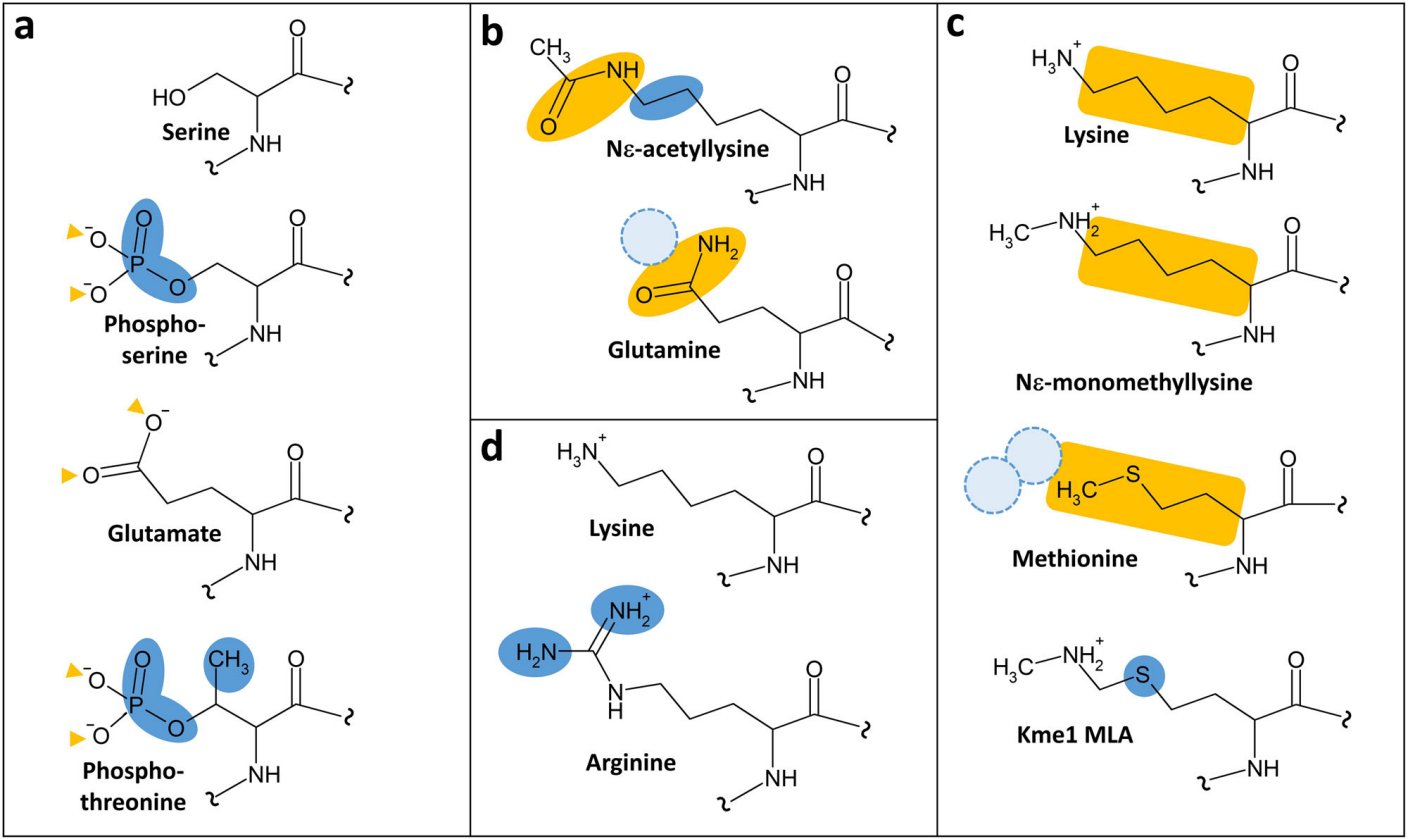
Comparison of the physicochemical properties of different natural and chemically modified amino acids. **a** Serine, phosphoserine, glutamate and phosphothreonine. **b** Acetyllysine and glutamine. **c** Lysine, monomethyllysine, methionine, and monomethyllysine analog (Kme1 MLA). **d** Lysine and arginine. Similarities are indicated in gold, differences in blue.

Focusing on protein lysine methylation (PKM) as an example, we summarize the options of target lysine replacements that can be employed in biochemical in vitro work and in cellular studies, evaluate if and to which extent they can be regarded as suitable mimetics of methylated or unmethylated lysine, and discuss how the challenges arising from the paucity of suitable amino acid mimics may be addressed. Of note, the conclusions drawn here also apply to the investigation of protein arginine methylation, which suffers from the same limitations.

## Requirements for faithful lysine methylation mimetics

Studies of protein lysine methylation typically address one or more of the following questions:

1. Is a specific lysine residue methylated by a certain protein lysine methyltransferase (PKMT)?
2. Does a substrate protein with a given methylation state (Kme0, 1, 2, or 3) of a defined lysine residue interact specifically with a certain reader domain?
3. Which characteristic cellular responses occur upon a lysine methylation or a methylation-specific binding event?

These questions can be approached in biochemical studies and in cellular experiments. Of note, Kme1, Kme2 and Kme3 are distinct states with specific (though sometimes partially overlapping) reader proteins and biological effects. Substrate proteins containing mutations, which mimic Kme0, 1, 2, or 3 would be powerful experimental tools. A mimic of unmethylated lysine should resemble lysine, but not be a substrate of the corresponding PKMT. To mimic the natural reader domain interactions, a Kme0 analog should be bound by Kme0 readers, but not by readers of Kme1/2/3. Conversely, mimics of Kme1, Kme2 or Kme3 (which we abbreviate as Kme1/2/3) should have the physicochemical properties of methyllysine in the corresponding methylation state, but they should be stable and interact only with their corresponding type of Kme1/2/3 readers, but not with all others and not with readers of Kme0.

## Target lysine replacements for experiments with PKMTs in vitro and in cells

General recommendations for biochemical and cellular studies to investigate the potential methylation of a lysine residue by a PKMT have been described elsewhere^9^. In such studies, K-to-A and K-to-R mutants of putative target lysine residues are useful to block the methylation. However, it needs to be identified if the lysine residue is the real target for methylation or if it is just involved in the PKMT interaction with the substrate peptide or protein. Of note, the target residue should be essential to obtain a methylation signal; residues mediating the enzyme-substrate interaction often are supportive, but methylation is not completely abrogated upon their exchange. If more than one methylated residue exists on the target protein, experiments are more complex and readout of methylation is best done by mass spectrometry.

K-to-M mutants at PKMT target sites have been established as inhibitors of PKMTs (Fig. 1c). This is structurally, biophysically, and chemically plausible and has been validated in numerous studies in vitro and in cells^10^. The side-chain of methionine is similar to the alkyl-chain of lysine, so this residue may occupy the hydrophobic lysine binding channel of PKMTs^11, 12^, though other binding modes have been observed as well. However, methionine is lacking the amino group, hence no enzymatic turnover is possible, leading to competitive inhibition of the enzyme. Based on this, inducible histone K-to-M mutations have been established as dynamic tools to probe the physiological role of site-specific histone methylation in cells and even in animal studies^13^.

## Target lysine replacements for biochemical in vitro characterization of reader domains

To study the methylation state-specific interaction of reader domains with substrate peptides or proteins biochemically, methylated lysine residues can be incorporated through chemical peptide synthesis into peptides and then semi-synthetically also into proteins^14^. This is the ideal experimental approach for biochemical in vitro studies. Alternatively, the methyllysine analog (MLA) strategy can be applied to prepare proteins site-specifically containing Kme1/2/3 MLAs^15^. In this approach, a single cysteine residue in the target protein is alkylated with a suitable compound to generate MLAs which differ from the methyllysine residues only by the exchange of one methylene group by a thioether (Fig. 1c). While this alteration increases polarity slightly, MLAs have been shown to interact with Kme1/2/3 readers in a specific manner in several cases^16^, though this needs to be validated experimentally for each reader domain. Conversely, mutating lysine to another natural amino acid is not a viable strategy to study reader domain binding, because K-to-A and K-to-R mutants can disrupt the interaction with Kme0 and Kme1/2/3 readers. Hence, they will not prevent binding in a methylation-specific manner. Moreover, amino acids that could trigger a positive and PKMT-independent interaction with Kme0/1/2/3 readers are not available.

## Target lysine replacements for studies of lysine methylation in cells

The lack of natural amino acids mimics of Kme0/1/2/3 particularly limits cellular studies as it precludes an approach analogous to the expression of S-to-E phosphomimetic target proteins. Previous papers (e.g^17, 18^.) used methionine and arginine to mimic Kme1 and Kme0, respectively, but this experimental strategy is not recommended. While arginine resembles lysine in its physicochemical properties, it is bulkier and has a different H-bonding capacity and geometry (Fig. 1d). Hence, a K-to-R exchange may disrupt reader protein interactions, if an unmethylated lysine is an important interface residue. Therefore, disruption of a reader protein interaction or biological signaling event by a K-to-R mutation cannot be taken as evidence that lysine methylation is involved. Furthermore, the presumption that a K-to-M mutant can mimic Kme1 is incorrect, because methionine does not resemble Kme1 in its critical biophysical properties (Fig. 1c). While the side-chain of methionine is similar to the alkyl chain of monomethyllysine, it is shorter and lacks both the positive charge and the positively polarized methyl-ammonium group including its H-bond donors. Hence, it is unlikely, that a reader protein specific for Kme1 interacts with methionine. Moreover, methionine is also quite different from unmodified lysine, because it lacks the amino group and the positive charge. Therefore, the formation or disruption of a protein-protein interaction in response to a K-to-M mutation does not give conclusive evidence for or against the involvement of the Kme1 methyl group in this binding event. Furthermore, mimics of Kme2 and Kme3 are lacking and discrimination of the effects of mono-, di- or trimethylation of lysine residues by mutational approaches is impossible.

## Recommended designs for cellular studies of lysine methylation

In addition to the obstacles described above, cellular PKM studies are further complicated by pleiotropic effects, because many PKMTs methylate more than one substrate protein and many reader domains interact with different methylated proteins as well. To show the direct effect of a specific protein methylation event on reader domain binding and identify downstream signaling effects in cells, one needs to conduct experiments with wild-type and PKMT KO cell lines together with wild-type methylation substrate and non-methylatable substrate mutants (K-to-R or K-to-A) in all combinations. Then, the effects directly mediated by lysine methylation of the target residue should have comparable impact on experimental signals in KO, K-to-R and combined KO/K-to-R cells. In contrast, signal changes observed only in KO or K-to-R cells, or changes that are elevated in combined KO/K-to-R cells, are likely mediated by other pathways. If the corresponding reader protein is known, reader domain mutants with disrupted binding pocket can be generated and included in these experiments as well. Another appealing alternative could be to incorporate methyllysine directly into the substrate protein as a non-natural amino acid during ribosomal protein synthesis^19^. This would offer a direct way to study the effects of methyllysine in the host cell but is experimentally challenging. Together, these experimental designs have the potential to lend a deeper understanding of the biological response connected to lysine methylation at a specific target site and identify PKMTs and reader proteins involved in the signaling process.

## References

[1] Blanc RS, Richard S (2017). Arginine Methylation: The Coming of Age. Mol. Cell.

[2] Luo M (2018). Chemical and Biochemical Perspectives of Protein Lysine Methylation. Chem. Rev..

[3] Husmann D, Gozani O (2019). Histone lysine methyltransferases in biology and disease. Nat. Struct. Mol. Biol..

[4] Cornett EM, Ferry L, Defossez PA, Rothbart SB (2019). Lysine Methylation Regulators Moonlighting outside the Epigenome. Mol. Cell.

[5] Jambhekar A, Dhall A, Shi Y (2019). Roles and regulation of histone methylation in animal development. Nat. Rev. Mol. Cell Biol..

[6] Biggar KK, Li SS (2015). Non-histone protein methylation as a regulator of cellular signalling and function. Nat. Rev. Mol. Cell Biol..

[7] Taverna SD, Li H, Ruthenburg AJ, Allis CD, Patel DJ (2007). How chromatin-binding modules interpret histone modifications: lessons from professional pocket pickers. Nat. Struct. Mol. Biol..

[8] Graves HK, Wang P, Lagarde M, Chen Z, Tyler JK (2016). Mutations that prevent or mimic persistent post-translational modifications of the histone H3 globular domain cause lethality and growth defects in Drosophila. Epigenet. Chromatin.

[9] Kudithipudi S, Jeltsch A (2016). Approaches and Guidelines for the Identification of Novel Substrates of Protein Lysine Methyltransferases. Cell Chem. Biol..

[10] Sahu V, Lu C (2022). Oncohistones: Hijacking the histone code. Annu Rev. Cancer Biol..

[11] Justin N (2016). Structural basis of oncogenic histone H3K27M inhibition of human polycomb repressive complex 2. Nat. Commun..

[12] Schuhmacher MK (2020). Sequence specificity analysis of the SETD2 protein lysine methyltransferase and discovery of a SETD2 super-substrate. Commun. Biol..

[13] Brumbaugh J (2019). Inducible histone K-to-M mutations are dynamic tools to probe the physiological role of site-specific histone methylation in vitro and in vivo. Nat. Cell Biol..

[14] Holt M, Muir T (2015). Application of the protein semisynthesis strategy to the generation of modified chromatin. Annu Rev. Biochem.

[15] Simon MD (2007). The site-specific installation of methyl-lysine analogs into recombinant histones. Cell.

[16] Jia G (2009). A systematic evaluation of the compatibility of histones containing methyl-lysine analogues with biochemical reactions. Cell Res.

[17] Yu B (2022). KMT5A-methylated SNIP1 promotes triple-negative breast cancer metastasis by activating YAP signaling. Nat. Commun..

[18] Farago A (2022). Acetylation State of Lysine 14 of Histone H3.3 Affects Mutant Huntingtin Induced Pathogenesis. Int J. Mol. Sci..

[19] Liu CC, Schultz PG (2010). Adding new chemistries to the genetic code. Annu Rev. Biochem.

